# Bacterial isolates and antimicrobial susceptibility patterns in tracheal wash samples from horses with equine asthma in Poland

**DOI:** 10.2478/jvetres-2026-0053

**Published:** 2026-09-26

**Authors:** Natalia Siwińska, Dorota Długopolska, Agnieszka Żak-Bochenek, Marzena Paszkowska, Paweł Rudzki, Ewa Kumiega, Anna Bogucka

**Affiliations:** Department of Internal Medicine and Clinic of Diseases of Horses, Dogs and Cats,; Department of Immunology, Pathophysiology and Veterinary Preventive Medicine,; Faculty of Veterinary Medicine, Wrocław University of Environmental and Life Sciences, 50-366 Wrocław, Poland; Vetlab, Polish Veterinary Laboratories, 52-017 Wrocław, Poland

**Keywords:** antimicrobial resistance, antimicrobial susceptibility, bacterial culture, equine asthma, tracheal wash

## Abstract

**Introduction:**

Equine asthma (EA) is a chronic inflammatory lower-airway disease. Bacterial growth from tracheal wash (TW) may occur in samples from horses with EA, but may represent colonisation rather than active infection. This study described cultivable bacterial isolates recovered from TW samples from horses with EA in Poland and assessed their antimicrobial susceptibility patterns and association with month/season of sampling and airway neutrophil percentage.

**Material and Methods:**

In total, 165 adult horses diagnosed with EA according to American College of Veterinary Internal Medicine–based criteria underwent endoscopy, TW sampling with a double-guarded catheter and bronchoalveolar lavage fluid cytology. The samples were cultured conventionally and their bacterial content identified with MALDI-TOF MS; bacteria were tested for antimicrobial susceptibility. Associations between culture results and categorical variables were analysed using chi-squared/Fisher-type tests with Monte Carlo simulation where appropriate; effect size was reported as Cramer’s V.

**Results:**

Culture results were positive in 117/165 horses’ samples (70.9%), and bacteria were isolated from 116/165 (70.3%). The most frequently recovered bacteria were *Streptococcus equi* subsp. *zooepidemicus*, *Pseudomonas aeruginosa* and *Klebsiella pneumoniae*. Culture positivity was not associated with month or season of sampling, whereas analysis of bacterial genus by month suggested a modest association (χ^2^ = 100.72, degrees of freedom = 77, P-value = 0.036; Cramer’s V = 0.305). No clear association was observed with airway neutrophil percentage. Antimicrobial susceptibility patterns varied by organism.

**Conclusion:**

Cultivable bacteria are frequently recovered from TW samples of horses with EA. Positive culture results should not be interpreted as evidence of bacterial infection without supporting clinical and cytological findings. Antimicrobial treatment, when clinically justified, should be guided by culture and susceptibility testing.

## Introduction

Equine asthma (EA) is a non-infectious chronic disease of the lower airways, characterised by recurrent exacerbations of clinical signs, including chronic cough, decreased exercise tolerance, nasal discharge and increased respiratory effort at rest or during exercise. Endoscopic examination combined with bronchoalveolar lavage fluid (BALF) cytology is considered a key component of EA diagnosis. Depending on clinical presentation and airway cytology, EA can be classified as mild/moderate or severe (8).

Mucus accumulation and impaired mucociliary clearance may favour bacterial colonisation of the lower airways; however, bacterial growth from tracheal wash (TW) samples is not synonymous with active bacterial infection. Bacteria have also been recovered from TW samples from clinically healthy horses, and culture-independent studies have shown that the equine respiratory tract contains complex microbial communities (1, 2, 25). Consequently, positive TW-culture results in asthmatic horses require cautious interpretation in the context of clinical signs, cytology and antimicrobial stewardship. Tracheal wash sampling is frequently used in clinical bacteriology because it can be performed during routine endoscopy and provides material from tracheal secretions, whereas BALF is primarily used for cytological confirmation and phenotyping of EA.

The aim of this study was to describe cultivable bacterial isolates recovered from TW samples from horses with EA in Poland and to assess their antimicrobial susceptibility patterns and association with month and season of sampling and airway neutrophil percentage. It was hypothesised that cultivable bacteria would be frequently recovered from TW samples of horses with EA, but that positive culture results would not necessarily indicate active bacterial infection. An additional objective of the study was to summarise the antimicrobial susceptibility patterns of the most frequently isolated bacteria to support culture-guided treatment decisions when secondary bacterial infection is clinically suspected.

## Material and Methods

### Horses.

With written consent from private owners, horses were enrolled which had been brought to the Wrocław University of Environmental and Life Sciences for endoscopic examination as part of EA diagnosis. Each animal underwent respiratory tract endoscopy, basic blood analysis, lung ultrasound and BALF cytology. Horses were included when they had not been treated for disease for at least two months before the planned sampling date and when they met American College of Veterinary Internal Medicine–based criteria for EA:

(a) one or more clinical signs of chronic respiratory disease (cough, nasal discharge or decreased performance) without fever;
(b) increased tracheal mucus accumulation (tracheal secretion accumulation score >1/5);
(c) BALF inflammation, defined in this study as neutrophils ≥10%, mast cells ≥2% or eosinophils ≥1%.

Horses that did not meet these criteria, horses with systemic disease or haematological evidence of infection, and horses with other causes of lung inflammation or reduced exercise capacity were excluded. Animals showing marked degenerative neutrophil changes in BALF were also excluded. Older horses with confirmed or suspected pituitary pars intermedia dysfunction were excluded to minimise potential confounding effects of endocrine disease on immune function and airway microbiological findings. The final study population comprised 165 adult horses of various breeds and both sexes, aged 6–18 years (mean 11.8 ± 3.4 years).

### Ethics committee approval.

Ethical evaluation and approval were not required for this study under the Law on Animal Experiments of January 15 2015 (Official Journal 2015, item 266) and the Amendment to the Law on Animal Experiments of November 17, 2021 (Official Journal 2021, item 2338) regarding the welfare of animals used for research or teaching purposes. Samples were taken as part of routine veterinary work and did not cause pain, suffering or stress equal to or greater than a needle puncture. All samples were obtained with written consent from the owner.

### TW samples.

Tracheal washing was performed during tracheoscopy. Airway endoscopy was performed under sedation using a combination of intravenous detomidine (Domosedan; Orion Pharma, Farmos Group, Turku, Finland) at 0.008–0.01 mg/kg and intravenous butorphanol (Morphasol; aniMedica, Senden-Bösensell, Germany) at 0.01–0.02 mg/kg with a 1.8-m fibre-optic videoendoscope (60332 PKS; Karl Storz, Tuttlingen, Germany). Tracheal mucus accumulation was scored from 0 to 5 as previously described (8, 9); a score greater than 1 was considered abnormal. Tracheal aspirates were collected approximately 10 cm proximal to the main carina using a double-guarded endoscopic catheter (double stage MILA Endoscopic Microbiology Aspiration Catheter; Mila International, Florence, KY, USA). Ten millilitres of sterile isotonic saline (0.9% NaCl) were infused and rapidly aspirated. Samples for culture were submitted to Vetlab Polish Veterinary Laboratories (Wroclaw, Poland) within 1 h. After TW collection, all horses underwent full endoscopic examination of the respiratory tract and BALF collection according to accepted standards using a BAL catheter (MILA Equine BAL Catheter 30FR, 300 cm) and 150 mL sterile prewarmed isotonic saline (0.9% NaCl). The BALF samples were used for cytological confirmation of EA, whereas TW was selected for bacterial culture because tracheal aspirates are routinely used for bacteriological assessment of lower airway secretions in clinical practice. Microbiological culture of BALF was not performed.

### Microbiological examination.

Before conducting the examination, TW samples were thoroughly mixed. Using a calibrated 10 µL loop, cultures were inoculated on agar media intended for bacterial growth, namely Columbia Blood Agar, Columbia CNA Agar, MacConkey Agar and Chocolate Agar (all Oxoid, Basingstoke, UK), which facilitate the growth of a broad range of microorganisms. For each test, a 0.5 mL volume of the TW samples was placed in Brain Heart Infusion Broth enrichment medium (Oxoid). Plates were incubated for 48 h at 37°C, and the Chocolate Agar was additionally incubated in a high CO_2_ atmosphere using a CO_2_Gen Compact system (Oxoid/Thermo Fisher Scientific, Basingstoke, UK). For anaerobic bacterial examination, cultures were also placed on Schaedler Anaerobe Agar with blood, and a 0.5 mL volume of TW sample was placed into Thioglycollate enrichment medium (both Oxoid). These cultures were incubated anaerobically for 4 d at 37°C with an AnaeroGen Compact insert (Oxoid/Thermo Fisher Scientific). Cultures for yeast-like fungi were established using YGC Medium (Oxoid). Plates were incubated for at least 3 d at 37°C. All plates were read every 24 h. If no growth was observed on solid agars but only turbidity in enrichment media, indicating microbial growth, broths (BHI and thioglycollate) were sub-cultured to identify growing microorganisms. In every case, direct preparations were also made from the sediment after centrifuging the TW samples at 500 rpm for 10 min. Slides stained by the Gram method were examined for the presence of yeast-like fungi and bacteria. Bacterial identification was conducted using a MALDI-TOF MS spectrometer (IV generation Sirius Biotyper, Bruker Daltonics, Bremen, Germany), which relies on protein spectrum analysis. Protein extraction followed the standard procedure recommended by the manufacturer (Bruker Daltonics), as described in a previous publication ( 27). Bacterial strains were applied onto a steel plate, and after drying, each sample was coated with 1 µL of α-cyano-4-hydroxycinnamic acid matrix solution. The obtained spectra were compared with the MBT Compass Library Revision K (Bruker Daltonics), which includes 4,274 species of bacteria and yeast-like fungi. Based on the manufacturer’s recommendations, the following score values were used to assess the reliability of the results: less than 1.7 indicated unreliable identification, a score of 1.7–2.0 allowed genus identification, a score of 2.0–2.3 provided secure genus identification and probable species identification, and a score above 2.3 indicated highly probable species identification. The assessment of aerobic bacterial susceptibility was conducted using the disc-diffusion method on Mueller–Hinton agar, Mueller–Hinton agar and blood or Mueller–Hinton agar with horse blood and nicotinamide adenine dinucleotide (all Oxoid), with the choice of agar plates dependent on the isolated microorganism. Plates were incubated under aerobic or microaerophilic conditions at 36°C, and the susceptibility evaluation was performed according to Clinical & Laboratory Standards Institute guidelines. For anaerobic bacteria, susceptibility testing was also carried out using the disc-diffusion method on fastidious anaerobe agar (Graso, Starogard Gdański, Poland), and minimum inhibitory concentration (MIC) values were determined using E-test strips and *Brucella* agar (Graso). Plates were incubated in anaerobic conditions at 36°C with the AnaeroGen Compact insert. The susceptibility assessment methods for anaerobic bacteria were tailored to the specific type of cultured microorganism. For *Prevotella* spp., *Bacteroides* spp., *Clostridium perfringens* and *Fusobacterium* spp., the disc-diffusion method was employed. For other species, susceptibility was evaluated based on the determination of MIC values for penicillin G and metronidazole. The susceptibility testing conformed to European Committee on Antimicrobial Susceptibility Testing guidelines. Bacterial growth was analysed as presence or absence with taxonomic identification; neither quantitative bacterial-load thresholds (in colony-forming units/mL) nor semi-quantitative categories of growth were applied. Therefore, low bacterial counts could not be separated from potentially clinically relevant bacterial burdens in the statistical analysis.

### Statistical analysis.

Descriptive statistics were used to summarise culture results, bacterial genera and species, month and season of sampling and antimicrobial susceptibility patterns. Percentages were calculated with explicit denominators: all horses, culture-positive horses, bacterial-positive horses or tested isolates, as appropriate. Associations between culture positivity and month or season were analysed using Pearson’s chi-squared test; when sparse counts were expected, Fisher-type exact testing or Monte Carlo simulation was used. The association between bacterial genus and month of sampling was analysed on the isolate level. Genera represented by at least five isolates were analysed separately, whereas rarer genera were grouped as “Other genera (<5)” to reduce fragmentation of categories. Effect size for contingency tables was reported as Cramer’s V. Associations between airway neutrophil percentage and culture result or number of isolates were assessed with non-parametric tests because neutrophil percentages were not assumed to be normally distributed. Antimicrobial susceptibility results were summarised descriptively by organism whenever possible; pooled resistance percentages were interpreted cautiously because antimicrobial panels and intrinsic susceptibility differed among bacterial species. Statistical significance was set at P-value < 0.05. Analyses were performed using spreadsheet-based summaries and statistical software capable of chi-squared and Monte Carlo procedures.

## Results

Positive culture results were recorded in samples from 117/165 horses (70.9%). After exclusion of the fungal isolate *Penicillium* spp. from the bacterial analyses, bacterial isolates were recovered in samples from 116/165 horses (70.3%). A total of 43 different microorganisms were recorded in the culture dataset. The distribution of isolates is presented in Table 1.

**Table 1.** Cultivable microorganisms isolated from tracheal wash samples of horses with equine asthma

Microorganism	Number of horses with bacteria after microbiological culture (direct)	Number of horses with bacteria after microbiological culture (after 48 h)	Number of horses with the given bacteria after both microbiological cultures
*Acinetobacter baumannii*	3	0	3
*Acinetobacter lwoffii*	1	0	1
*Acinetobacter radioresistans*	1	0	1
*Actinobacillus* spp.	2	0	2
*Actinobacillus equuli*	2	1	3
*Actinobacillus rossii*	3	0	3
*Actinobacillus suis*	2	0	2
*Alcaligenes faecalis*	1	0	1
*Bacteroides fragilis*	2	0	2
*Bordetella bronchiseptica*	1	1	2
*Burkholderia cepacia*	1	0	1
*Burkholderia* spp.	1	0	1
*Clostridium perfringens*	1	0	1
*Clostridium tertium*	1	0	1
*Enterobacter hormaechei*	2	1	3
*Enterobacter ludwigii*	1	0	1
*Escherichia coli*	2	0	2
Lactase^⁻^ *Escherichia coli*	2	0	2
*Klebsiella aerogenes*	1	0	1
*Klebsiella oxytoca*	5	0	5
*Klebsiella pneumoniae*	11	0	11
*Kosakonia cowanii*	1	0	1
*Ochrobactrum anthropi*	1	0	1
*Pantoea agglomerans*	2	0	2
*Pasteurella* spp.	3	0	3
*Penicillium* spp.	1	1	2
*Prevotella* sp.	1	0	1
*Pseudomonas aeruginosa*	27	0	27
*Pseudomonas azotoformans*	1	0	1
*Pseudomonas fulva*	1	0	1
*Pseudomonas gessardii*	1	0	1
*Pseudomonas putida*	3	0	3
*Pseudomonas* spp. other than *P. aeruginosa*	7	0	7
*Pseudomonas synxantha*	1	0	1
*Rhodococcus equi*	2	0	2
*Serratia marcescens*	2	0	2
*Shewanella putrefaciens*	2	0	2
*Shigella* spp.	1	1	2
Coagulase^⁻^ *Staphylococcus*	1	0	1
*Staphylococcus sciuri* subsp. *sciuri*	0	1	1
*Staphylococcus aureus*	2	0	2
*Staphylococcus epidermidis*	0	1	1
*Staphylococcus xylosus*	1	0	1
*Stenotrophomonas maltophilia*	6	0	6
α-haemolytic *Streptococcus*	1	0	1
*Streptococcus canis*	1	0	1
*Streptococcus dysgalactiae*	2	0	2
*Streptococcus dysgalactiae* subsp. *equisimilis*	1	0	1
*Streptococcus equi* subsp. *zooepidemicus*	33	2	35

The most frequently isolated bacterium among bacterial-positive cultures was *Streptococcus equi* subsp. *zooepidemicus* (n = 35; 30.2% of bacterial-positive cultures), followed by *Pseudomonas aeruginosa* (n = 27; 23.3%) and *Klebsiella pneumoniae* (n = 11; 9.5%). Bacterial growth was obtained from direct culture in 111/116 bacterial-positive samples (95.7%), whereas bacterial growth was noted in five samples only after enrichment/incubation. Mixed bacterial growth was observed in 39/116 bacterial-positive cultures (33.6%), with more than one bacterium isolated.

The distribution of culture-positive and culture-negative TW samples by month is shown in Fig. 1. Culture positivity was not significantly associated with the month of sampling (χ^2^ = 7.65, degrees of freedom (df) = 11, P-value =0.745; Cramer’s V = 0.215) or with season (χ^2^ = 0.59, df = 3, P-value = 0.898; Cramer’s V = 0.060). For the exploratory isolate-level analysis of bacterial genus by month, 155 bacterial isolates were included after excluding *Penicillium* spp., entries without a bacterial isolate and isolates without a recorded month. When genera represented by fewer than five isolates were grouped as “Other genera (<5)”, a statistically significant but modest association between bacterial genus and month of sampling was observed (χ^2^ = 100.72, df = 77, asymptotic P-value = 0.036; Monte Carlo P-value = 0.036; Cramer’s V = 0.305). Because the contingency table was sparse, this result should be interpreted as exploratory. The distribution of the main bacterial genera by month is presented in Fig. 2.

**Fig. 1.**
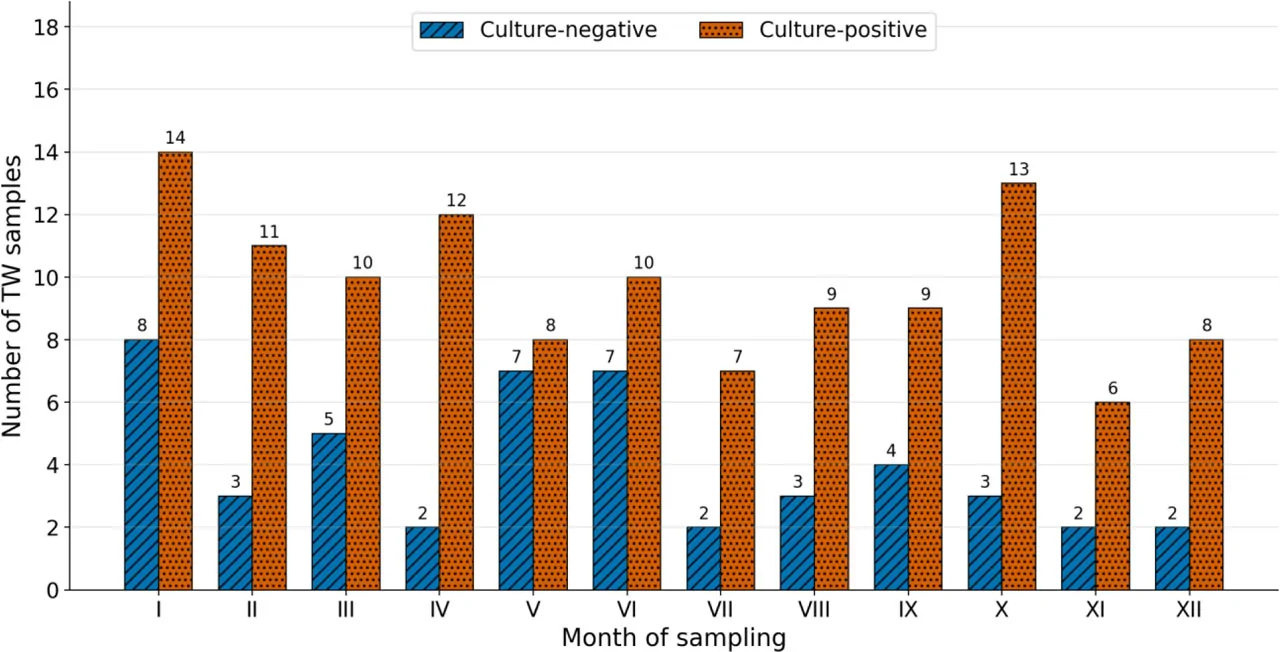
Number of culture-positive and culture-negative tracheal wash samples from horses with equine asthma

**Fig. 2.**
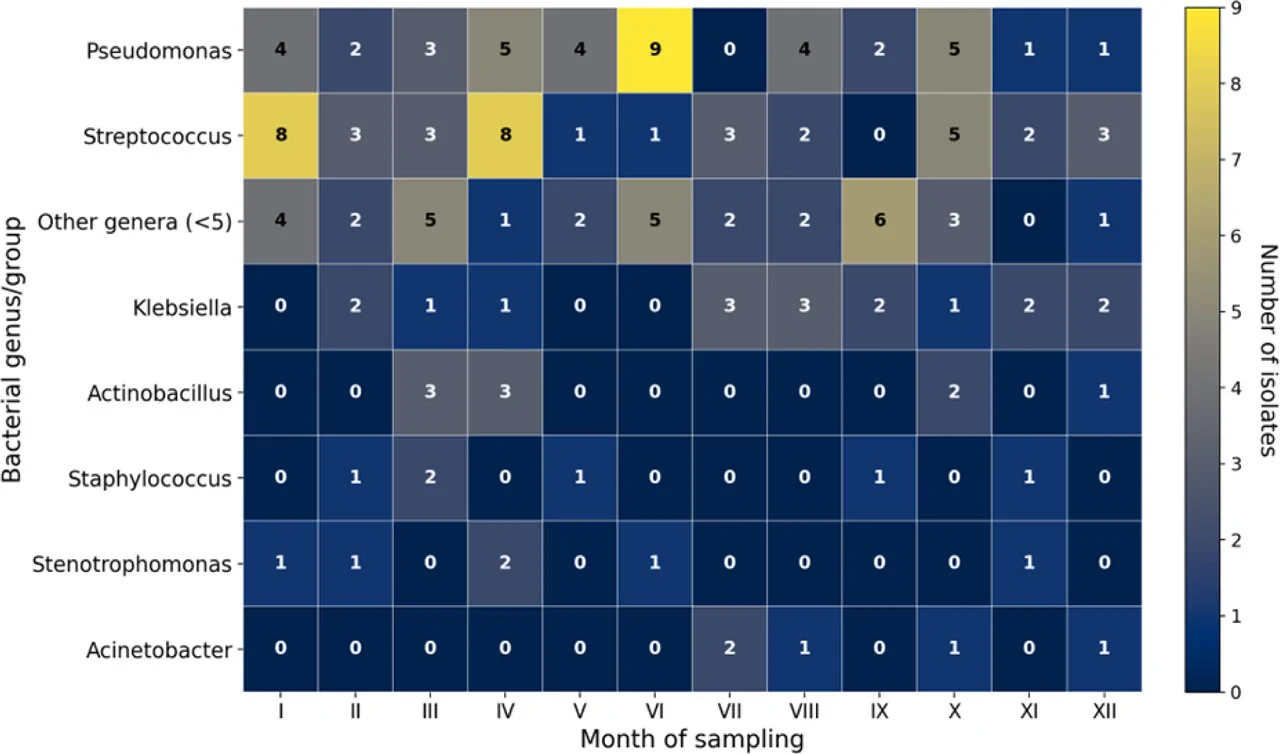
Distribution of the main bacterial genera in tracheal wash samples from horses with equine asthma

Antimicrobial susceptibility results varied substantially by organism and by antimicrobial tested. Because not all antimicrobials were tested against all isolates, and because intrinsic susceptibility differs among bacterial species, pooled resistance values in Table 2 should only be interpreted descriptively and not as species-level resistance rates. The highest pooled resistance proportions were observed for rifampicin, cephalexin, cefuroxime and ticarcillin with clavulanic acid; however, these values were based on heterogeneous bacterial species and different denominators. Species-level patterns for the most frequently isolated bacteria are summarised in Table 3. No clear association was observed between bacterial culture result or number of isolates and airway neutrophil percentage (P-value = 0.4). The results are presented in Fig. 3.

**Table 2.** Antimicrobial substances tested against isolates from tracheal wash samples from horses with equine asthma

Active substance	Number of cultures showing resistance	Total number of cultures challenged with the active substance	Percentage of cultures showing resistance
Amikacin	8	71	11.3
Cephalexin	27	32	84.4
Cefuroxime	25	32	78.1
Ceftiofur	21	105	20
Cephquinome	21	108	19.4
Erythromycin	46	94	48.9
Enrofloxacin	14	137	10.2
Gentamicin	24	95	25.3
Marbofloxacin	12	135	8.89
Penicillin G	49	108	45.4
Rifampicin	31	35	88.6
Trimethoprim + sulfamethoxazole	40	124	32.3
Ciprofloxacin	1	5	20
Polymyxin B	0	5	0
Colistin	0	2	0
Tobramycin	3	12	25
Clindamycin	10	10	10
Amoxicillin with clavulanic acid	3	6	50
Amoxicillin	9	32	28.1
Ampicillin	17	69	24.6
Ceftazidime	5	37	13.5
Cefepime	0	2	0
Metronidazole	1	5	20
Piperacillin	0	2	0
Sulfadiazine + trimethoprim	14	86	16.3
Doxycycline	10	46	21.7
Azithromycin	9	56	16.1
Oxytetracycline	2	25	8
Tetracycline	27	63	42.9
Cephapirin	2	37	5.4
Cefazolin	6	41	14.6
Ticarcillin with clavulanic acid	10	18	55.6
Streptomycin	6	17	35.3
Pefloxacin	0	1	0
Florfenicol	0	1	0

**Table 3.** Antimicrobial agents to which the greatest susceptibility and resistance was found among the bacteria most frequently isolated from horses with equine asthma

Bacterial species	Antimicrobial to which the bacteria was susceptible	Antimicrobial to which the bacteria was resistant
*Streptococcus equi* subsp. *zooepidemicus*	ceftiofur (n = 35)	tetracycline (n = 18)
	cephquinome (n = 35)	
	erythromycin (n = 35)	
	enrofloxacin (n = 22)	
	marbofloxacin (n = 28)	
	sulfamethoxazole/trimethoprim (n = 29)	
	ampicillin (n = 29)	
	sulfadiazine/trimethoprim (n = 33)	
	penicillin G (n = 34)	
	azithromycin (n = 35)	
	cephapirin (n = 27)	
	cefazolin (n = 27)	
*Pseudomonas aeruginosa*	amikacin (n = 24)	gentamicin (n = 11) enrofloxacin (n = 9)
	gentamicin (n = 15)	
	marbofloxacin (n = 19)	
	ceftazidime (n = 21)	
*Klebsiella pneumoniae*	ceftiofur (n = 10)	single resistant strains to different antimicrobials
	cefquinome (n = 10)	
	enrofloxacin (n = 8)	
	marbofloxacin (n = 9)	
	gentamicin (n = 9)	
	sulfamethoxazole/trimethoprim (n = 8)	
	sulfadiazine/trimethoprim (n = 6)	

**Figure 3.**
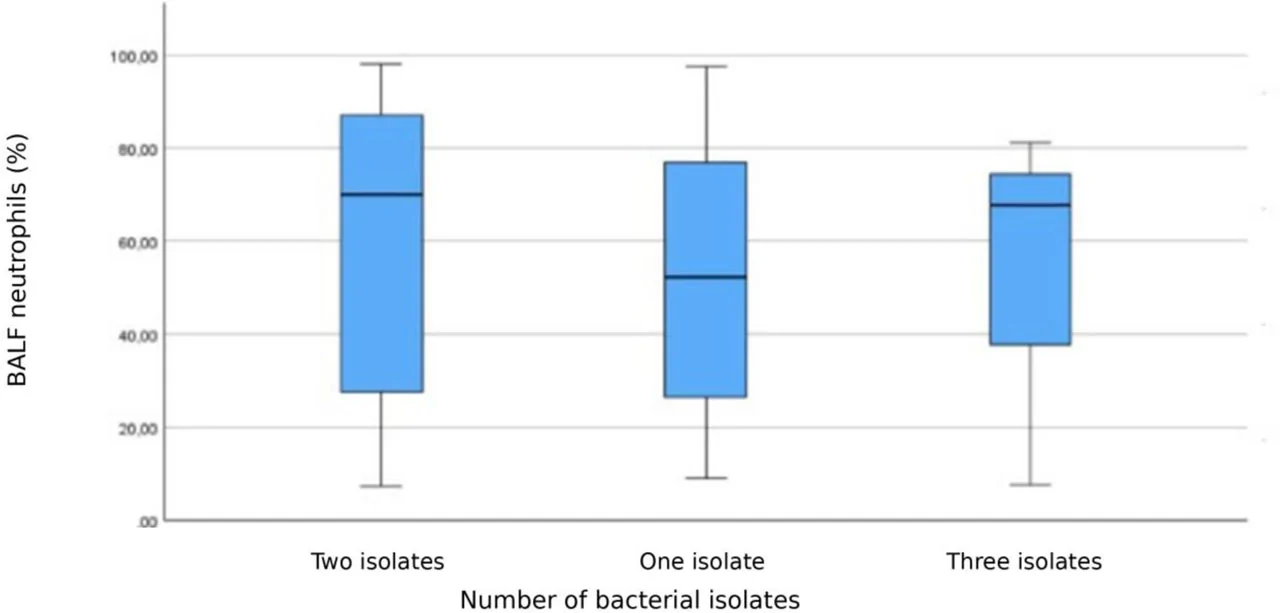
Airway neutrophil percentage according to the number of bacterial isolates recovered from tracheal wash samples from horses with equine asthma

## Discussion

This is the first study conducted in Poland to describe cultivable bacterial isolates recovered from TW samples of horses with EA and evaluate their antimicrobial susceptibility. The study demonstrated that positive culture results from tracheal fluid are common in horses with EA. However, because a healthy control group was not included, these findings should be interpreted as descriptive clinical data and cannot establish whether the recovered bacteria are causally associated with EA.

In humans, even healthy lower airways are not sterile and contain a resident microbiota. Studies on suboptimally controlled asthma have shown that higher airway bacterial diversity and altered community composition are significantly associated with a greater degree of bronchial hyperresponsiveness, suggesting that the airway microbiota structure may contribute to asthma heterogeneity (16, 30).

In the present study, a substantial proportion of horses with EA had a positive TW culture. Manguin *et al.* (25) similarly reported bacterial growth in 56% of aspirates from horses with moderate asthma, although the cohort was smaller. These findings indicate that bacterial growth in the tracheal fluid of asthmatic horses is a common finding and may reflect airway colonisation or impaired clearance. Importantly, bacteria can also be cultured from the TW samples of clinically healthy horses (1, 25), because the equine respiratory tract contains resident bacterial communities (2). Therefore, positive TW cultures should not be interpreted as evidence of bacterial infection without supporting clinical, cytological and, where available, quantitative microbiological information.

In a finding consistent with that of Manguin *et al.* (25), *Streptococcus* spp. were the most frequently isolated bacteria. In our cohort, *S. equi* subsp. *zooepidemicus* was the most common individual bacterial taxon. In racehorse populations with lower airway disease, the reported prevalence of *S. equi* subsp. *zooepidemicus* ranged from about 5–6% in some cohorts to 16–38% in others, indicating that this bacterium is a frequent isolate in affected horses (5, 34). This bacterium has also been isolated from TW samples of healthy horses, supporting the possibility that it may behave as a commensal or opportunistic coloniser depending on host and environmental conditions (1, 25). In the absence of a control group, our data do not demonstrate overgrowth relative to healthy horses. In racehorses, *Streptococcus* spp. in tracheal samples have been associated with respiratory signs, mucus accumulation and increased neutrophil proportions (6), whereas this relationship was not observed in the older cohort studied by Manguin *et al.* (25). In our study, a standardised clinical severity score was not available for all horses, and no clear association was observed between bacterial category and airway neutrophil percentage.

Besides *Streptococcus equi* subsp. *zooepidemicus*, the most frequently isolated bacteria were *Pseudomonas aeruginosa* and *Klebsiella pneumoniae*. This pattern contrasts with that observed in a previous study in asthmatic horses, in which isolates were predominantly *Actinobacillus* spp. and other Pasteurellaceae (25). In a study in France of horses with pneumonia, *P. aeruginosa* was isolated from respiratory samples, whereas *K. pneumoniae* was reported among adult horses with bacterial pneumonia in the USA (10, 14). Differences between findings may reflect variations in geography, horse populations, sampling methods, prior management, environmental exposure and the limitations of standard culture methods in recovering fastidious organisms. Because only culture-based methods were used, the present study should not be interpreted as a complete characterisation of the tracheal microbiota. Some organisms often considered potential contaminants may also belong to the normal equine airway flora: Proteobacteria, Actinobacteria, Firmicutes and Bacteroidetes are major phyla reported in the equine respiratory tract, and *Staphylococcus* spp., *Bacillus* spp. and unidentified Gram-negative bacterial genera have been reported in healthy horses (2, 25).

Bacterial presence and growth in TW or BALF have previously been associated with an increased likelihood of degenerate neutrophil morphology compared with culture-negative samples, and age may modify this relationship (17). In our study, marked degenerative neutrophil changes were an exclusion criterion and the degree of neutrophil degeneration was not analysed as an outcome. We also did not identify a clear association between culture result, bacterial category or number of isolates and airway neutrophil percentage. This supports a cautious interpretation: bacterial recovery from TW samples in horses with EA may represent colonisation or impaired clearance rather than active bacterial infection.

Seasonal variation in airway microbiology has been reported in human respiratory diseases, including chronic obstructive pulmonary disease and paediatric respiratory infections (4, 28). Other studies have described seasonal variation in colonisation by specific organisms, such as higher summer frequency of *K. pneumoniae* colonisation in asymptomatic healthcare workers, although the mechanisms remain unclear (15). To date, seasonal patterns in equine TW or BALF culture results have been insufficiently investigated. In our cohort, culture positivity was not associated with month or season of sampling. The exploratory isolate-level analysis suggested a modest association between bacterial genus and month, with combinations such as *Acinetobacter* and July, *Pseudomonas* and June and other rare genera and September contributing most to the overall chi-squared statistic. However, because many genera were rare and the contingency table was sparse, this finding should be interpreted as one generating a hypothesis rather than one providing confirmation.

Antimicrobial resistance is a serious and growing global public health problem that hampers the successful control and treatment of bacterial infections. Excessive and inappropriate antimicrobial use contributes to the selection of resistant bacteria, including the extended-spectrum beta-lactamase-producing *Escherichia coli*, methicillin-resistant *Staphylococcus aureus* and multidrug-resistant *Salmonella* which have been reported in horses (31). Tracheal wash cultures from horses with EA mainly revealed isolates’ susceptibility to commonly used antimicrobial agents, but selected organisms’ resistance to several agents was also observed. This should be considered clinically relevant when antimicrobial therapy is contemplated. Resistance patterns should be interpreted with caution, because different susceptibility testing methods, interpretive criteria and organism-specific intrinsic resistance profiles affect susceptibility testing results (13). The most clinically useful interpretation is, therefore, at species-level rather than in aggregate. *Streptococcus equi* subsp. *zooepidemicus* isolates in this study showed susceptibility to several commonly used drugs, but resistance to tetracycline was frequent. Previous equine studies have generally reported susceptibility of *S. zooepidemicus* to penicillin, although resistance to other selected antimicrobials has been described in different regions (7, 12, 23, 24).

In equine practice, aminoglycosides are widely used to treat *P. aeruginosa* infections in horses (19). In our horses with EA, most *P. aeruginosa* isolates were susceptible to amikacin, marbofloxacin and ceftazidime, whereas their susceptibility to enrofloxacin and gentamicin was variable. This supports the need for culture and susceptibility testing before treatment. Resistance reported for agents such as ampicillin, chloramphenicol, tetracyclines and trimethoprimsulphonamide should be interpreted cautiously, as it may have limited clinical relevance as a reflection of intrinsic resistance rather than constructive in therapy selection as an indication of acquired resistance (18). Susceptibility patterns vary across studies: resistance to gentamicin and/or enrofloxacin has been reported (3) and Léon *et al.* (22) found resistance to cefquinome, gentamicin and marbofloxacin; however, high susceptibility to amikacin, enrofloxacin, and gentamicin has been noted in other equine isolates (26).

Therapeutic protocols for *K. pneumoniae* infections in horses often include ceftiofur, gentamicin, potentiated sulphonamides, enrofloxacin, doxycycline or chloramphenicol (11, 33). Although the *K. pneumoniae* isolates from our horses with EA were largely susceptible to the tested antimicrobials, studies from other countries have described widespread resistance, including multidrug-resistant and ESBL-producing strains, in both healthy and diseased horses (14, 20, 29, 32). These findings reinforce that culture and susceptibility testing rather than reliance on empirical treatment practices should guide antimicrobial selection.

The main limitation of the present study is the absence of a control group of clinically healthy horses. Therefore, the study cannot determine whether the recovered bacteria are more frequent in horses with EA than in healthy horses, nor can it establish causal or pathogenetic relationships. The decision to focus exclusively on horses with EA was made because inclusion of a comparable number of healthy control horses would have required invasive sampling that was not feasible for ethical reasons. Another limitation concerning the cohort is that clinical severity was not assessed with a standardised score for all horses, and potentially relevant environmental factors, including bedding, forage type, stable ventilation, dust exposure, transport history and details of previous antimicrobial exposure were not available for analysis. A limitation related to the procedures on samples is that BALF was used for cytology but was not submitted for microbiological culture; therefore, direct comparison between TW and BALF bacteriology was not possible. What was cultured did not have bacterial load thresholds (CFU/mL) or semi-quantitative growth categories recorded, preventing us from distinguishing low-level colonisation from potentially clinically relevant bacterial burdens. Additionally, only standard culture methods were used, so non-cultivable or fastidious bacteria may have been missed. Therefore, the results should not be equated with the complete airway microbiota. Finally, the antimicrobial susceptibility panels were not uniform for every bacterium, and some antimicrobials were tested on small numbers of isolates; pooled resistance rates should therefore be interpreted cautiously. Future studies should include healthy controls when ethically feasible, standardised clinical severity scoring, environmental sampling, bacterial-load estimation and culture-independent microbiome approaches.

## Conclusion

Cultivable bacteria were frequently recovered from TW samples of horses with EA in Poland, with *Streptococcus equi* subsp. *zooepidemicus*, *Pseudomonas aeruginosa* and *Klebsiella pneumoniae* being the most common bacterial isolates. Culture positivity was not associated with month or season of sampling, and no clear relationship was observed with airway neutrophil percentage. Exploratory isolate-level analysis suggested a modest association between bacterial genus and month, but this finding should be interpreted cautiously because of the sparsity of the categorical data. Positive TW culture results should not be interpreted as evidence of bacterial infection without supporting clinical and cytological findings. Antimicrobial treatment, when clinically justified, should be guided by culture and susceptibility testing to support antimicrobial stewardship.
